# Practical geospatial and sociodemographic predictors of human mobility

**DOI:** 10.1038/s41598-021-94683-7

**Published:** 2021-07-28

**Authors:** Corrine W. Ruktanonchai, Shengjie Lai, Chigozie E. Utazi, Alex D. Cunningham, Patrycja Koper, Grant E. Rogers, Nick W. Ruktanonchai, Adam Sadilek, Dorothea Woods, Andrew J. Tatem, Jessica E. Steele, Alessandro Sorichetta

**Affiliations:** 1 Population Health Sciences, College of Veterinary Medicine, Virginia Tech, Blacksburg, VA USA; 2 WorldPop, School of Geography and Environmental Science, University of Southampton, Southampton, UK; 3 Google, Mountain View, CA USA

**Keywords:** Environmental social sciences, Risk factors

## Abstract

Understanding seasonal human mobility at subnational scales has important implications across sciences, from urban planning efforts to disease modelling and control. Assessing how, when, and where populations move over the course of the year, however, requires spatially and temporally resolved datasets spanning large periods of time, which can be rare, contain sensitive information, or may be proprietary. Here, we aim to explore how a set of broadly available covariates can describe typical seasonal subnational mobility in Kenya pre-COVID-19, therefore enabling better modelling of seasonal mobility across low- and middle-income country (LMIC) settings in non-pandemic settings. To do this, we used the Google Aggregated Mobility Research Dataset, containing anonymized mobility flows aggregated over users who have turned on the Location History setting, which is off by default. We combined this with socioeconomic and geospatial covariates from 2018 to 2019 to quantify seasonal changes in domestic and international mobility patterns across years. We undertook a spatiotemporal analysis within a Bayesian framework to identify relevant geospatial and socioeconomic covariates explaining human movement patterns, while accounting for spatial and temporal autocorrelations. Typical pre-pandemic mobility patterns in Kenya mostly consisted of shorter, within-county trips, followed by longer domestic travel between counties and international travel, which is important in establishing how mobility patterns changed post-pandemic. Mobility peaked in August and December, closely corresponding to school holiday seasons, which was found to be an important predictor in our model. We further found that socioeconomic variables including urbanicity, poverty, and female education strongly explained mobility patterns, in addition to geospatial covariates such as accessibility to major population centres and temperature. These findings derived from novel data sources elucidate broad spatiotemporal patterns of how populations move within and beyond Kenya, and can be easily generalized to other LMIC settings before the COVID-19 pandemic. Understanding such pre-pandemic mobility patterns provides a crucial baseline to interpret both how these patterns have changed as a result of the pandemic, as well as whether human mobility patterns have been permanently altered once the pandemic subsides. Our findings outline key correlates of mobility using broadly available covariates, alleviating the data bottlenecks of highly sensitive and proprietary mobile phone datasets, which many researchers do not have access to. These results further provide novel insight on monitoring mobility proxies in the context of disease surveillance and control efforts through LMIC settings.

## Introduction

Human populations are connected now more than ever in human history, with worldwide travel booming over the last century^1^. Understanding these mobility patterns across subnational spatial and temporal scales has important implications across science, from urban planning and infrastructure development to disease transmission^2,3^. Seasonal international and domestic mobility patterns and corresponding changes in population dynamics are difficult to capture in temporally-static surveys or censuses^4^, yet have crucial implications for vector-borne and respiratory infectious diseases^1,5,6^, as highlighted by the most recent coronavirus global pandemic^7^. Seasonality and subsequent changes in mobility can further impact and exacerbate disease outbreaks and epidemics^4^, including cholera, measles, malaria, Zika virus, influenza, etc.^2,4,8–10^, resulting from increased travel during holidays or mass gatherings. More recently, emergent changes in population mobility throughout 2020 due to non-pharmaceutical interventions (e.g. lockdowns) aimed at curbing the COVID-19 pandemic have further highlighted the need to understand seasonal population movement patterns and measure mobility quickly and comprehensively^1,7,11^.

Human movement is generally characterized by frequent trips travelled over short distances, interspersed with longer distances which occur more occasionally^3,12^. A number of factors may influence these trips, ranging from short-term movements due to factors such as job and family priorities^3^, to seasonal migration driven by cyclical climatic variations^2,13^, to long-term migration and displacements resulting from catastrophes and natural disasters^4^. Quantifying human mobility patterns over highly resolved spatial and temporal scales, however, has proven difficult, owing to a lack of sufficient spatially-resolved datasets spanning long periods of time^1,3,8,14^. Historically, studies have used high spatial resolution satellite imagery data such as night-time lights to assess temporal variations in population densities^2^, but these data are only a proxy for quantifying population metrics^4^. Traditional mobility data such as travel history surveys^15^, traffic surveys^16^, migration surveys and censuses^17^ have been used to more directly quantify population movements spanning both short and long-term movements, but such surveys and censuses are typically not spatially resolved, and/or can further suffer from limitations such as recall bias. Alternatively, studies using global positioning system (GPS) trackers can provide insight into highly localized movement patterns^18^, but are typically worn for only short periods of time, such as weeks or months, among participants representing a sample size which may not be representative of national and international populations^14,19^. Recently, researchers are increasingly employing call data records (CDRs) to explore population movements at both high spatial and temporal scales^4^, where mobile phones collect locational information from cell phone towers whenever a billable event (such as a call or text message) occurs. These datasets therefore offer locational data at relatively high temporal and spatial resolutions, with the latter depending upon a country’s cell phone tower infrastructure, but again suffer from limitations including the inability to track cross-border movements and a lack of data for time periods between billable events^14,19^.

Over recent years, passively collected data from mobile phone technology has filled a novel niche in quantifying human mobility patterns at high spatial resolutions, across international borders, and spanning wide temporal periods such as years^1^. The Google Aggregated Mobility Research Dataset (GAMRD) represents one such form of passively collected data, aggregated over users who have turned on the Location History setting, which is off by default. The applicability of this data has been shown to be comparable with more traditional forms of mobility signals, such as travel history surveys^14,20^. While these data are likely biased in terms of wealth, gender, and urbanicity^21,22^, mobile penetration rate and smartphone ownership within sub-Saharan Africa have been steadily increasing over the decades, with nearly half a billion people currently subscribing to mobile services and an estimated 65% of the population having access to a smartphone device by 2025^22^. More recently, GAMRD data have further shown to be strongly predictive of human mobility patterns as measured through call data records, with a Pearson correlation coefficient nearing 0.90 between Google data and Vodafone mobility data for parts of Europe^7^. These data have proven timely and responsive in quickly informing reductions in mobility and social contacts as a result of non-pharmaceutical interventions, such as lockdown and social distancing measures, in the context of the 2019 coronavirus disease (COVID-19) pandemic^7,23^. These studies suggest that while GAMRD data are subject to similar sociodemographic biases inherent to mobile phone data, they are highly accurate and representative of movement patterns captured among the study population.

Despite its utility in describing population movement patterns, exploiting these large mobility datasets may be challenging for many researchers due to restrictive data sharing policies put in place to protect individual privacy^9^. This often necessitates the use of freely available proxy data, such as satellite-derived imagery or modelled socioeconomic surfaces to approximate changes in population densities, yet few studies have explored the ability of these correlates to explain seasonal population movements^2^. The need for publicly available data that can be used to study mobility patterns has become more apparent as a result of the COVID-19 pandemic. Researchers have emergently explored mobility patterns using proprietary mobile datasets, and found that although mobility patterns reduced globally as a result of the pandemic, the extent of these reductions varied both by country and pandemic waves^11^. Corporations across the world have recognized the utility in understanding the effectiveness of restricted mobility in preventing subsequent pandemic waves, and have increasingly begun to release openly available data for researchers to more easily study mobility patterns, including Facebook, Apple, Google, and OpenSky^24^. Towards this, understanding how openly available socioeconomic and geographic datasets explain mobility patterns pre-pandemic is vital to establish a baseline for understanding how mobility changed as a result of the pandemic, as well as how mobility might be permanently altered once the pandemic subsides.

Here, we aim to address this knowledge gap by (1) measuring pre-pandemic seasonal patterns of domestic and international population movements using GAMRD data from smartphones, using Kenya as a case study, and (2) explaining population mobility patterns using a suite of geospatial and socio-economic covariates within a spatiotemporal Bayesian framework. Specifically, we firstly describe overall monthly changes in population movements across 2018 and 2019, including differences in between-country, within-country, and within-county movements. Secondly, we utilise a hierarchical Bayesian modelling approach to identify key correlates of overall monthly movement patterns while accounting for spatial and temporal autocorrelations.

## Methods

### Study area

**Figure 1:**
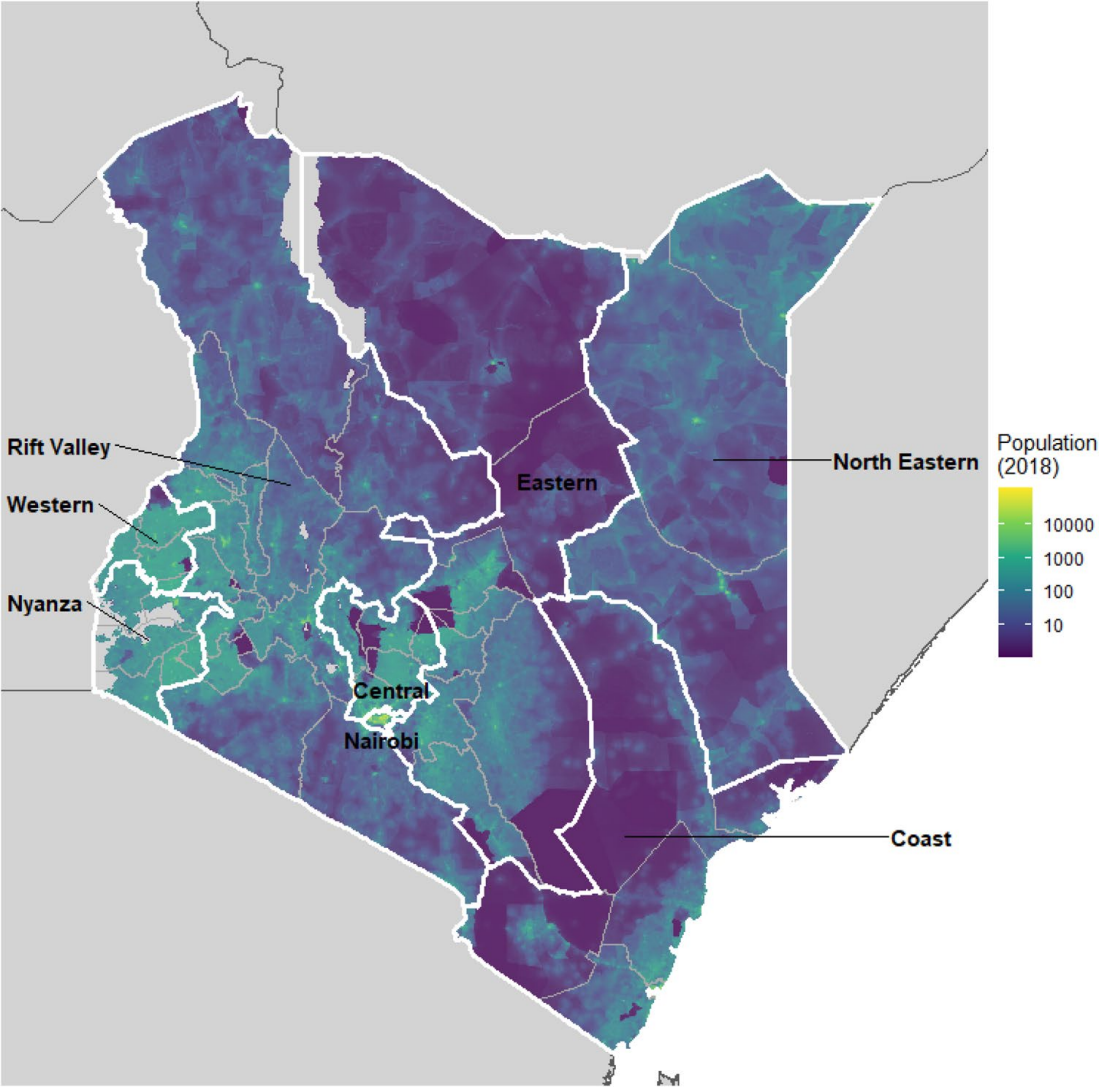
Provinces (labelled, white, n = 8) and counties (grey, n = 47) in Kenya, with population counts per km^2^ as of 2018. R software, version 4.0.4, https://www.r-project.org/.

As of 2019, Kenya had a population of 52.5 million people, with a population density of 92.4 per km^2^^25^. According to data collected through the Malaria Indicator Survey conducted through the Demographic and Health Surveys Program, over 90% of households within Kenya owned a mobile telephone in 2015, ranging from 85% in the eastern portion of Kenya to almost 95% in the central portions of Kenya containing urban areas like Nairobi^26^. Of note, Kenya adopted a new form of geographic administration in 2013 as a result of the Constitution of 2010, where administrative divisions were devolved into 47 counties (e.g., administrative I level)^27^. While the analyses performed here were conducted at the county level, internal movement patterns are also presented at the provincial level to facilitate broad mobility patterns across the country. Figure 1 shows the 47 counties and 8 provinces used in these analyses, as well as the estimated population distribution in 2018, as gathered through WorldPop (https://www.worldpop.org/)^28,29^.

### Human mobility data

The Google Aggregated Mobility Research Dataset contains anonymized mobility flows aggregated over users who have turned on the Location History setting, which is off by default. This is similar to the data used to show how busy certain types of places are in Google Maps—helping identify when a local business tends to be the most crowded. The dataset aggregates flows of people from region to region, which is here further aggregated at county, province, and country levels. In 2016, this dataset represented over 300 million users from nearly all countries around the world, with sufficient data to cover ~ 40% of the world’s population^30^.

To produce this dataset, machine learning is applied to logs data to automatically segment it into semantic trips^31^. Briefly, location data points are triangulated using a variety of GPS, WiFi and cell phone tower signals, and converted to trips using a variety of signals, including timing and location of data points (e.g., stopping at airports when travelling internationally), dwell times, and other factors^30,32^. These trips have been shown to be spatially equivalent to GPS data within 100 m, and capture more international movements, where traditional mobile phone operators may lack location data across international borders^14^. Regardless, this approach is limited by the user’s data availability; if, for example, a user tends to frequently turn their location services on and off (for example to save phone battery), this may lead to biased results for that individual. To provide strong privacy guarantees, all trips were anonymized and aggregated using a differentially private mechanism^33^ to aggregate flows over time (see https://policies.google.com/technologies/anonymization). Briefly, this process provides privacy guarantees by (1) bounding the posterior probability that any individual’s data was used in producing an output; and (2) only flows representing a sufficiently large sample size (e.g., greater than 100 devices) are processed within the model^30^. This research is done on the resulting heavily aggregated and differentially private data. No individual user data was ever manually inspected, only heavily aggregated flows of large populations were handled.

All anonymized trips are processed in aggregate to extract their origin and destination location and time. For example, if users travelled from location a to location b within time interval t, the corresponding cell (a, b, t) in the tensor would be n ∓ err, where err is Laplacian noise. The automated Laplace mechanism adds random noise drawn from a zero mean Laplace distribution and yields ($\epsilon$, δ)-differential privacy guarantee of $\epsilon$ = 0.66 and δ = 2.1 × 10^−29^ per metric. Specifically, for each week W and each location pair (A,B), we compute the number of unique users who took a trip from location A to location B during week W. To each of these metrics, we add Laplace noise from a zero-mean distribution of scale 1/0.66. We then remove all metrics for which the noisy number of users is lower than 100, following the process described in^33^, and publish the rest. This yields that each metric we publish satisfies (ε,δ)-differential privacy with values defined above. The parameter $\epsilon$ controls the noise intensity in terms of its variance, while δ represents the deviation from pure $\epsilon$-privacy. The closer they are to zero, the stronger the privacy guarantees.

This dataset represents monthly domestic mobility flows at the county level in Kenya over the course of 2018–2019, in addition to international mobility flows. We further explored the different types of movement within these data, comprised of within-county travel (where the origin and destination counties remained the same); domestic travel (where the origin county differed from destination county, but was still within Kenya); and international travel (where the destination was outside of Kenya). Figure S1 shows these types of movement patterns across months (combined for 2018 and 2019), as represented by box plots. Within-county trips (blue) across shorter distances tended to comprise the majority of travel across months, followed by domestic travel (red) and international travel (green). Similar datasets have been described further in^7,23,30^.

To measure seasonal movement patterns across the time period, we quantified the change in monthly population flows. This change was calculated for each month as the total outward mobility flow from each source county to all domestic and international destinations (denoted as *monthly mobility flows)*, which was compared to January of the corresponding year (denoted as the *baseline mobility flow*). Of note, January represents an arbitrary baseline, chosen for its intuitive comparison of mobility patterns throughout the rest of the calendar year. We then calculated the monthly change in population flows as a ratio for each subsequent month (February through December) by dividing the *monthly mobility flows* by the corresponding *baseline mobility flow* of the equivalent year. For example, if the average outward population flow out of Nairobi for February 2019 was double the average outward population flow for January 2019, the change in mobility would be quantified as 2. Values close to 1 therefore represent no change in mobility patterns for that month as compared to the January *baseline mobility flow*, while values greater than 1 represent an increase in mobility and values less than 1 represent a decrease in mobility. This is represented by the following equation,

$$\Delta y_{i, Feb, Mar,Apr \ldots Dec} = \frac{{x_{i,Feb, Mar,Apr \ldots Dec} }}{{x_{i,Jan} }}$$

where *y* represents the change, $\Delta$, in mobility for a given county, *i*, and *x* represents total outward mobility flow at the county level. These calculations result in a dataset with n = 1128 observations, representing 24 data points for the months of January through December across 2018 and 2019, multiplied by 47 counties within Kenya.

### Covariate data

We used a combination of socioeconomic and geospatial covariates to explore the change in mobility patterns over time. Table 1 shows the covariates used in these models, as well as sources, temporal coverage, and native spatial resolution.

**Table 1:** Covariate data, sources, spatial resolution, and temporal resolution/coverage.

Covariate	Range (SD)	Native spatial resolution	Temporal resolution	Temporal coverage	Source
% population living in urban extent	0.001–0.947 (0.175)	1 km^2^	Year	2015	GHS-SMOD^34^
% people living in povertyy^1^	0.099–0.917 (0.185)	1 km^2^	Year	2008	WorldPop^35^
% women with no primary education	0.032–0.17 (0.03)	5 km^2^	Year	2017	Local Burden of Disease^36^
Travel time (minutes) to the nearest urban centre^2^	4.37–380.66 (98.92)	1 km^2^	Minutes	2015	Malaria Atlas Project^37^
# school holidays (days)	0–31 (9.8)	National	Month	2018–2019	Lai et al.^38^
Aridity index	96.65–223.1 (20.8)	30 arc-seconds		1970–2000	Global Aridity Index and Potential Evapotranspiration Climate Database v2^39^
Enhanced Vegetation Index (EVI)	1325–4914 (838.4)				MODIS^40^
Precipitation (mm)^3^	1.70–266.9 (55.85)	2.5 arc-minutes		2010–2018	WorldClim v2^41^
Temperature (°C)^3^	14.7–28.8 (3.17)				
VIIRS Night-time lights	0.246–15.27 (1.96)	15 arc-seconds (~ 500 m^2^)		2012–2018	NOAA/NGDC Earth Observation Group^42^

^1^Poverty is defined as proportion of residents living in MPI-defined poverty.

^2^Urban centre defined as a contiguous area with 1500 or more inhabitants per square kilometre, or a majority of built-up land cover coincident with a population centre of at least 50,000 inhabitants.

^3^Available at: https://worldclim.org/data/monthlywth.html.

Socioeconomic covariates used at the county level in these analyses included the proportion of population living in urban areas, the proportion of the population living in poverty, proportion of women with no primary level education, accessibility to the nearest urban centre, and the number of school holidays over 2018 and 2019. These covariates were included in our analyses as mobile phone ownership and use have been shown to be biased towards wealthier populations, which in turn correlates with increased education and urbanicity^43^. Further, the distance travelled and number of trips taken have been shown to vary by sociodemographic factors, including wealth and education^30,37^. Yet despite socioeconomic biases in mobile phone ownership, mobility patterns as estimated through mobile phone data have proven robustly representative of population-level movements, specifically within Kenya^43^.

Geospatial and climatic covariates used in these analyses included aridity, temperature, precipitation, an enhanced vegetation index (EVI), and night-time lights. Briefly, aridity represents a measure of climatic moisture, while EVI is a measure of vegetation greenness. The night-time lights dataset quantifies light emission during the night-time at the daily level, which has been used as a measure of dynamic population density. These covariates have been shown to correlate and predict population-level movements, both at the global scale and within sub-Saharan Africa^2,30^. Specifically, climatic variables such as aridity, precipitation and EVI have been shown to correlate with agricultural and crop seasons, where seasonal labour migration occurs^44,45^, while temperature can correlate with increased travel due to seasonal holidays, including school holidays^46^ and religious holidays such as Christmas. Lastly, night-time lights have been shown as an important proxy for seasonal fluctuations in population density, suggesting human movements into and out of cities^2^.

The proportion of the Kenyan population living within urban extents was calculated using the Global Human Settlement (GHS) Settlement Model grid (SMOD)^47^, classifying settlement typologies into Degree of Urbanization (e.g., ‘rural’, ‘peri-urban’, and ‘urban’ clusters)^47–49^. Peri-urban and urban clusters were combined to form ‘urban’ versus ‘rural’ extents, which was applied to 2018 and 2019 population data, as obtained through WorldPop^29^. The population living within urban clusters was therefore quantified, and divided by total population within the county to estimate the proportion of population within the county living within an urban extent. The proportion of population living in poverty at a 1 km^2^ spatial resolution was also obtained via WorldPop^35^, and averaged across counties to obtain the proportion of population living in poverty per county. The proportion of women having no primary level education at a spatial resolution of 5 km^2^ was obtained via the Local Burden of Disease project^36^, while accessibility to the nearest urban centre (as measured via travel time) was obtained via the Malaria Atlas Project^37^, and both were similarly averaged across counties. Lastly, the number of school holidays within Kenya over the course of 2018 and 2019 was obtained via Lai et al.^46^, capturing the number of country-specific holidays observed among primary and secondary schools for the corresponding year.

Monthly aridity was obtained from the Global Aridity Index and Potential Evapotranspiration Climate Database v2^39^, while precipitation and temperature data were obtained from the WorldClim v2 datasets^41^, representing mean aridity, precipitation and temperature at the county level for each month from 2012 through 2018, thereby reducing yearly fluctuations in estimates. The aridity index can be used to “quantify precipitation availability over atmospheric water demand”, representing a confluence of precipitation, temperature and evapotranspiration processes, which measures the ability of the atmosphere to remove moisture^39^. Monthly enhanced vegetation indices were obtained from the Moderate Resolution Imaging Spectroradiometer (MODIS) Vegetation Index Products available through the National Aeronautics and Space Administration^40^, measuring vegetation greenness through satellite derived imagery. These similarly represent mean EVI for each month over the course of 2012 through 2018, which have been shown to correlate with seasonal movements as a proxy for crop seasons^44,45^. Lastly, Visible Infrared Imaging Radiometer Suite (VIIRS) data were obtained at a spatial resolution of 15 arc-seconds (or approximately 500 m at the equator) through the National Oceanic and Atmospheric Administration and National Geospatial Data Clearinghouse’s Earth Observation Group^42^. These data detect electric lighting on the Earth’s surface and can be used to quantify night-time lights^50^, with implications for changes in population densities^2^. Data were similarly obtained for months between 2012 and 2018, and averaged at the county level.

### Statistical modelling framework

In these analyses, we aimed to infer whether a suite of covariates have a scientifically meaningful and statistically significant relationship with human mobility, as quantified through the GAMRD, representing an explanatory modelling analysis^51^. To do this, we utilized a Bayesian spatiotemporal modelling approach within the R-INLA package^52^ to investigate seasonal population mobility patterns using a suite of geospatial and socio-economic covariates while accounting for spatial and temporal autocorrelation inherent in the mobility data. Similar models have been used previously to describe spatiotemporal dynamics in malaria prevalence and incidence^53,54^, as well as geographic heterogeneity in subnational mortality rates due to sparse events such as suicide and lung cancer across the USA^55,56^. By employing a spatiotemporal Bayesian framework, both fixed effects and spatially structured and unstructured random effects can be added to the model, as well as time-dependent covariates and effects, and interactions between space and time variables^52,53,55,57^, allowing for exploration of patterns at the spatial, temporal, and spatiotemporal level^53^.

The general structure of the Bayesian spatiotemporal model utilized in these analyses can be defined as^57^:

$$\log \left( {y_{it} } \right) = \beta_{0} + A_{i} + B_{t} + C_{it} + X_{it}^{^{\prime}} \beta + \epsilon_{it}$$

where $y_{it}$ represents the change in population movement in county *i* at time *t*, where a log transformation was performed to satisfy Gaussian assumptions; $\beta_{0}$ represents the model intercept; $A_{i}$ represents the spatial autocorrelation component, modelled using a *bym2* family structure; $B_{t}$ represents the fixed a effects in the model, while incorporating the temporal component, *t*, modelled with an *ar1* structure; $C_{it}$ represents the interaction between the spatial and temporal components $A_{i}$ and $B_{t}$; and $X_{it}^{^{\prime}}$ represents the spatially and time-varying covariates in the model (described above), $\beta$ represents the regression parameters^53^, and $\epsilon_{it}$ represents the Gaussian error term.

## Results

**Figure 2:**
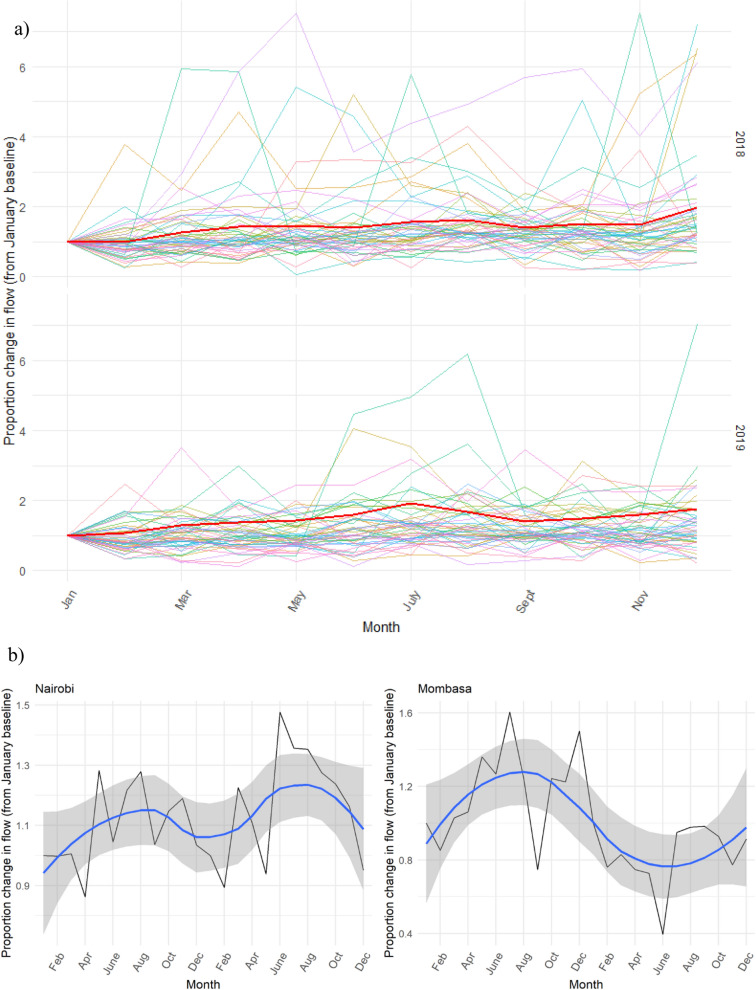
(**a**) Observed change in monthly population flows for 2018 and 2019, as compared to the baseline mobility flow in January of the corresponding year. Coloured lines represent seasonal mobility patterns for each county within Kenya, and the red line represents the average seasonal mobility pattern for all counties over the year. (**b**) Observed mobility patterns (as shown in the top panel) among selected counties, overlaid with smoothed Loess curves.

Figure 2 shows monthly mobility flows, across 2018 and 2019, in all Kenyan counties (Fig. 2a) and in Nairobi and Mombasa (Fig. 2b), which comprise a large proportion of Kenya’s population. In Fig. 2a, coloured lines represent monthly mobility flows for each of the 47 counties within Kenya, while the darker red line represents the overall mean of monthly mobility flows across all counties. Overall, monthly mobility flows varied between 0.672 in Mandera county to 7.515 in Samburu county (mean 1.424; SD 1.061) for 2018, and 0.110 in Tana River county to 25.866 in Elgeyo-Marakwet county (mean 1.464; SD 2.18) for 2019. Movement patterns in Fig. 2b represent mobility flows for Nairobi (left) and Mombasa (right) counties, and further show mobility flows using Loess smoothing, as indicated by the dark blue line and grey confidence intervals. This smoothing method employs smoothed conditional means, and is useful in visualizing seasonal mobility patterns devoid of monthly noise. Using this smoothing technique among these two counties clearly outlines the variability observed in movement patterns between populous counties. For example, in Nairobi mobility tended to increase and peak in late summer for both 2018 and 2019, while in Mombasa, inverse patterns were observed across both years, with a peak in mobility in June of 2018 but a trough in June of 2019.

Figure S3 outlines international destinations from Kenya across 2018 and 2019, where mobility flows were log transformed to better highlight spatial variability. Unsurprisingly, four of the top five international destinations observed fell within Africa, with the top three destinations comprised of Tanzania, Uganda and Ethiopia, respectively, within Eastern Africa. Other top destinations included Middle Eastern hubs such as the United Arab Emirates and Qatar, South Africa, and European countries including Great Britain, France and the Netherlands.

**Figure 3:**
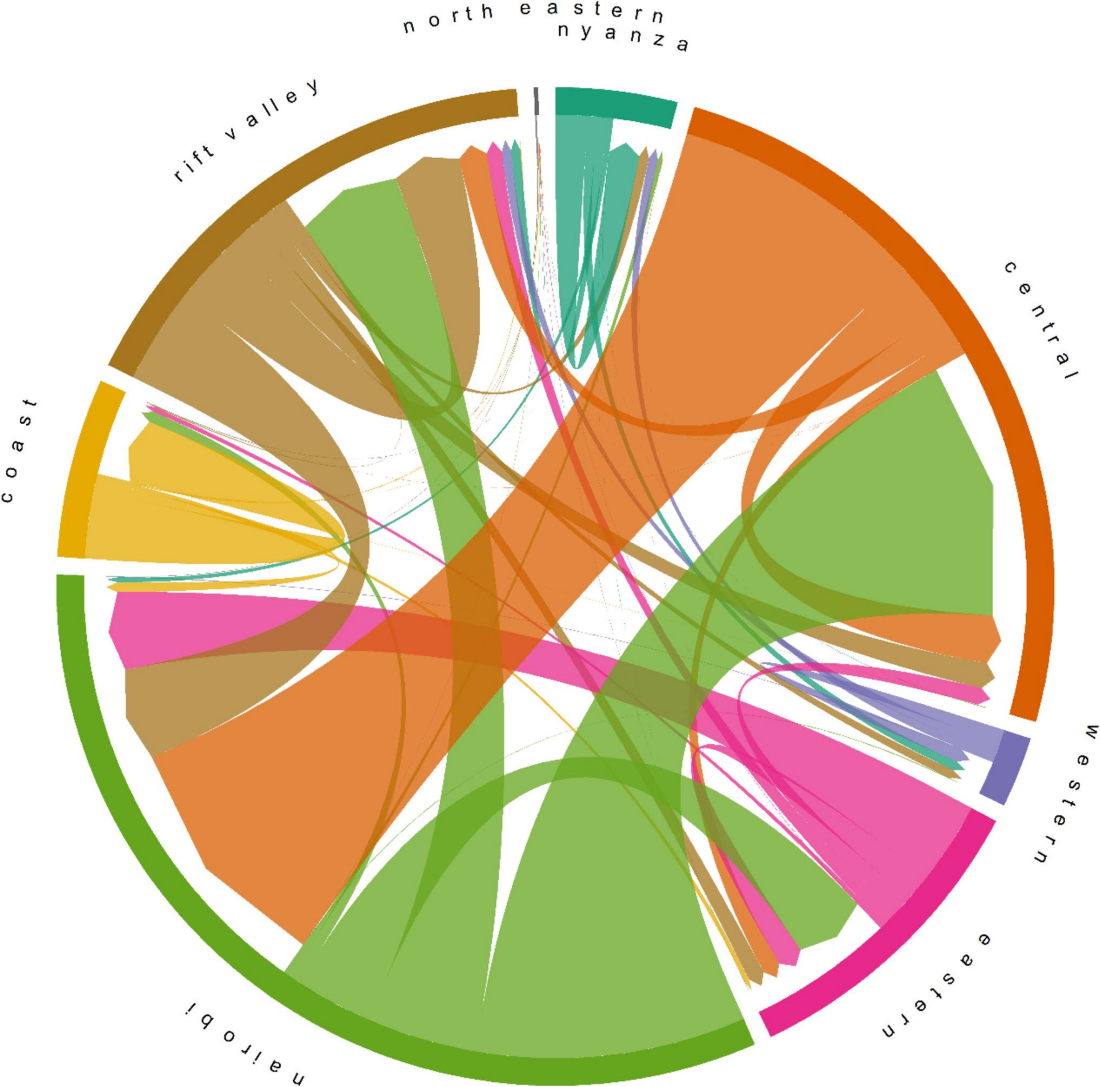
Bidirectional domestic mobility flows between Kenya provinces. Arrows indicate directionality of movement.

We similarly explored domestic travel destinations within these datasets, as represented in Fig. 3. This circle plot represents bidirectional movement between Kenyan counties, broadly grouped by provinces (Figure S2). For example, green represents movement corresponding to Nairobi, with arrows representing proportional outward movement from Nairobi to other regions such as the Central and Rift Valley provinces. This means that roughly half of outward movement originating within the Nairobi province travelled to the Central province, while approximately one-quarter of outward movement travelled to the Rift Valley province, and another quarter to the Eastern province. Conversely, nearly half of the inward movement to Nairobi consisted of movements originating within the Central province (orange), while similarly a quarter of inward flows originated in the Rift Valley (brown) and Eastern (pink) provinces.

### Model results

**Figure 4:**
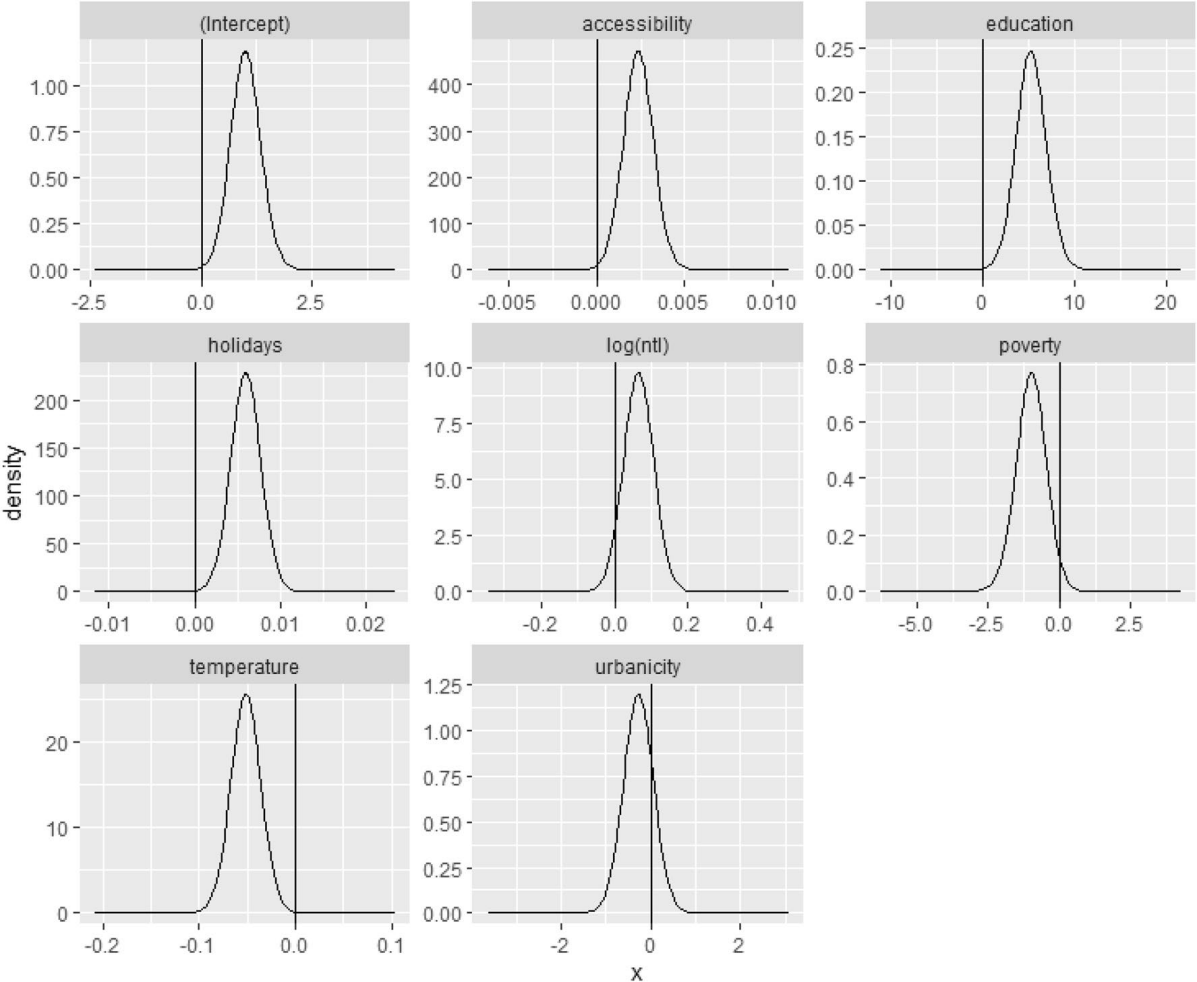
Posterior densities of modelled fixed effects. Parameters with peak densities further away from zero indicate a stronger influence on the model. *NTL* night-time lights.

Lastly, we aimed to identify key socioeconomic and geospatial correlates of monthly mobility flows by employing a Bayesian modelling framework. Table 2 shows posterior marginal effects for the fixed effects within the model, as well as model hyperparameters. Lower and upper 95% credible intervals for fixed model effects are also shown, with values crossing 0 indicating no significant effect within the model. Peak density estimates of posterior distributions among fixed effects included in the model are shown in Fig. 4. The further peak densities are from zero, the more influence the effect has on the model^58^.

**Table 2:** Marginal effects of the fixed effects and hyperparameters of the posterior distributions, plus model statistics.

Parameter	Mean	Lower 95% CI	Upper 95% CI
Intercept	1.037	0.369	1.756
Accessibility to urban centre	0.002	0.001	0.004
Education	5.337	1.996	8.867
School holidays	0.007	0.004	0.01
Night-time lights	0.04	− 0.036	0.117
Poverty	− 1.098	− 2.25	− 0.017
Temperature	− 0.052	− 0.082	− 0.022
Urbanicity	− 0.236	− 0.913	0.437

	Mean	SD	95% CI
**Hyperparameters**			
Precision_Gaussian_	3.838	0.0167	0.655
Precision_Spatial_	12.875	3.35	13.087
Precision_Temporal_	18,791.484	1.20e + 5	1.28e + 5
φ	0.209	0.163	0.599
ρ_Spatial_	0.995	0.005	0.002
ρ_Temporal_	− 0.229	0.524	1.757
DIC	1744.04		
P_D_	59.39		
Marginal likelihood	− 528.36		
**Model validation**			
MAE	0.573		
RMSE	1.532		
Psuedo-R^2^	0.203

**Figure 5:**
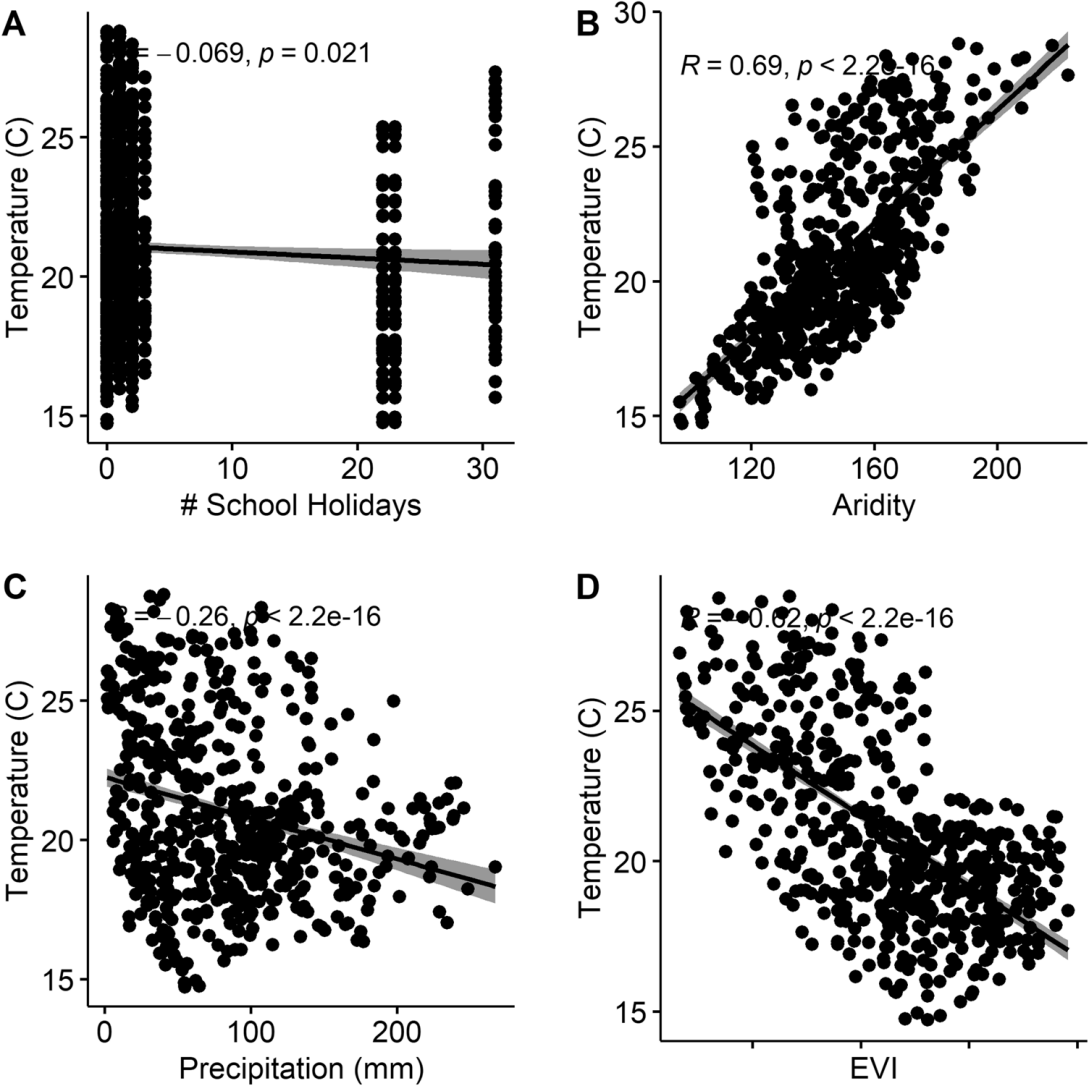
Correlation plots between (**a**) temperature (°C) and number of school holidays per month; (**b**) temperature (°C) and aridity; (**c**) temperature (°C) and precipitation (mm); and, (**d**) temperature (°C) and enhanced vegetation index.

We firstly explored the correlation between geospatial covariates to investigate potential multi-collinearity. We found that aridity, EVI, and precipitation were strongly correlated with temperature (Fig. 5), and therefore included only temperature in the model to improve model fit. We found that accessibility to the nearest urban centre, education, number of school holidays, and temperature had significant effects on the model. Specifically, greater accessibility to urban centres, higher education, and greater number of school holidays explained increased mobility, where decreasing temperature explained less mobility (Fig. 4). Night-time lights and poverty had slight effects on the model, but 95% CI estimates overlapped with zero, suggesting these effects were not significant. Lastly, model validation statistics are reported, including mean absolute error (MAE), root mean square error (RMSE) and pseudo-R^2^ values. MAE and RMSE represent model precision and bias, with values closer to zero representing better model fit, while pseudo-R^2^ values represent variance explained by the model^59,60^.

**Figure 6:**
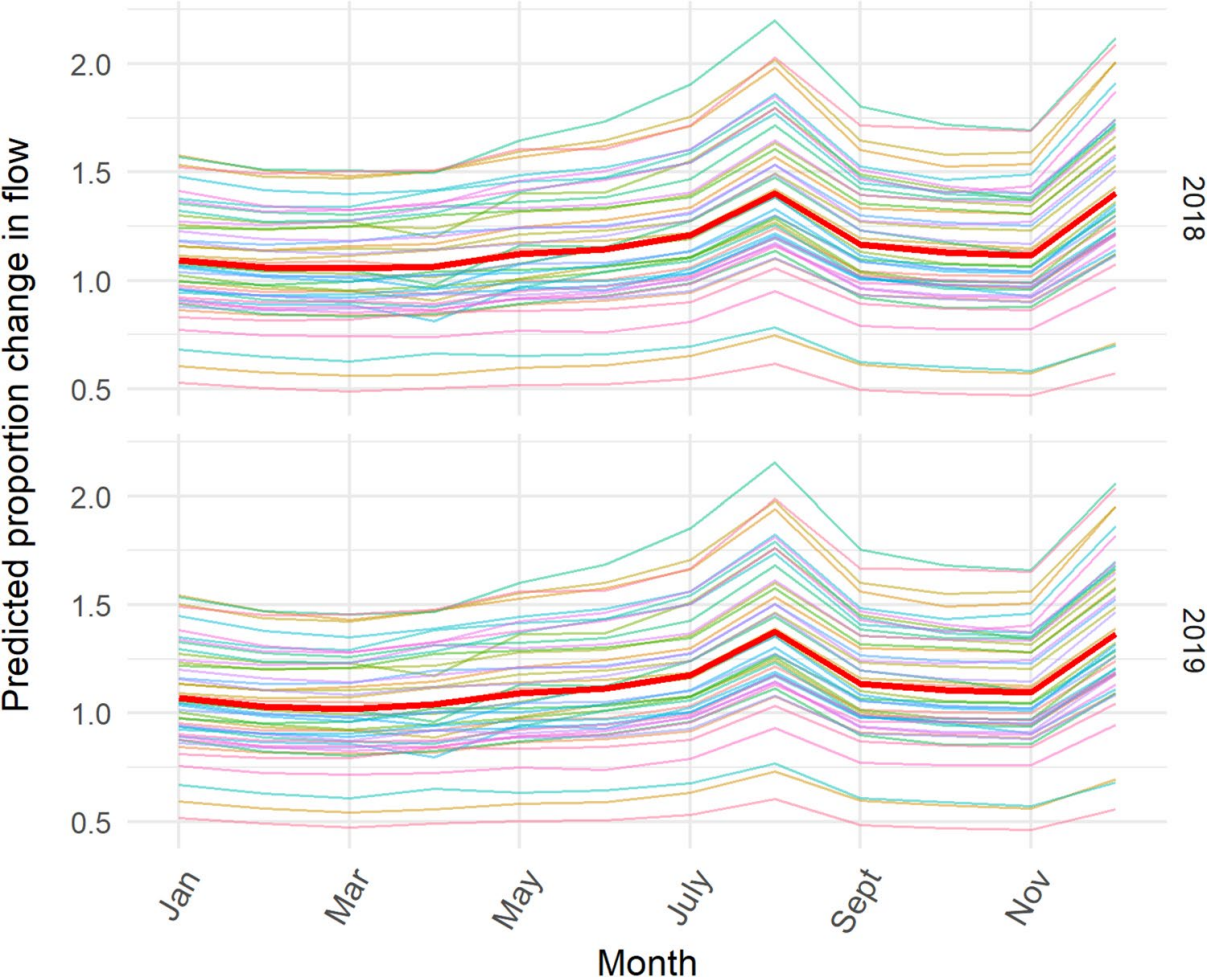
Modelled monthly mobility flows for 2018 and 2019, as compared to the baseline mobility flow for January. Coloured lines represent seasonal mobility patterns for each county within Kenya, and the red line represents the average seasonal mobility pattern for all counties over the year.

Finally, we visualised mobility as a result of the spatiotemporal model used, presenting ‘smoothed’ estimates of observed mobility patterns for 2018 and 2019 (Fig. 6), representing overall mobility flows devoid of noise. Similar to Fig. 2a, these estimates represent the monthly mobility flows, as compared to a January baseline mobility flow for the corresponding year. Coloured lines represent monthly mobility flows for each county within Kenya, while the red line represents the average monthly mobility flow across all counties. Throughout both years, mobility tended to decrease in February, and increase throughout the spring and summer months, peaking in August. A sharp decrease in mobility was observed in the fall months before preceding another increase in mobility around the holidays in December.

## Discussion

Establishing baseline mobility patterns and understanding the proxies and correlates of population movements across space and time are fundamental across a variety of disciplines, from infrastructure planning to disease surveillance^30^. However, such analyses have rarely been performed with a robust dataset spanning many years at high spatial resolutions, and have timely implications, given the recent COVID-19 global pandemic. Taking Kenya as an example, here, we address this research gap using a novel mobile phone dataset by establishing monthly mobility patterns across multiple years and exploring key sociodemographic and geospatial correlates of this mobility. We found that a set of commonly available covariates can capture these patterns well, offering the possibility of estimating and mapping seasonal mobility in LMIC settings.

We found that the majority of trips tended to occur along shorter distances within counties, while longer distance trips between counties and across countries occurred less frequently within the dataset (Figure S1), in line with other studies^1,14,30^. We further found that between-county and between-country trips tended to occur between spatially proximate counties and countries, with the majority of domestic mobility comprised of movements between Nairobi and Kiambu counties (Central province), Kajiado county (Rift Valley province), and Machakos county (Eastern province), comprising nearly 75% of the overall movement into Nairobi (Figure S4). These patterns were unsurprising, given the close spatial proximity of these counties to each other (Figure S2). We similarly found the majority of international mobility destinations were other East African countries, such as Tanzania, Uganda, and Ethiopia, again suggesting that these countries are highly interlinked by mobility. These results have particular implications for disease surveillance and monitoring efforts within the context of the COVID-19 pandemic, where coordination between counties and countries will be vital in stemming further regional outbreaks and resurgent waves^7^.

Lastly, we identified key correlates of mobility across years, and found that socioeconomic variables including urbanicity, poverty, and female education strongly explained mobility patterns, in addition to geospatial covariates such as accessibility to major urban centres and temperature. We found that the number of school holidays significantly explained mobility patterns, with greater mobility occurring on months with a higher number of school holidays, such as August. However, our model validation results suggest that the observed changes in mobility patterns over the course of the study period are only partly explained by the covariates selected in our model, with a pseudo-R^2^ value of 0.203, suggesting other individual level socioeconomic covariates (for example access to transportation or occupation) might be important to explore in future analyses. As these analyses involved anonymized mobility data at the county level, individual level demographics linked to mobility patterns were not able to be included in our model. Regardless, while the socioeconomic factors used are likely subject to general biases in mobile phone ownership, such as wealth and urbanicity^43^, previous studies have found that mobility estimates from such datasets still robustly represent population-level movement, further suggesting that these variables are necessary to include in any model exploring human movement.

Temperature was found to explain mobility in our analyses, and was further not statistically correlated with school holidays, as determined by a pairwise t-test with Bonferroni correction (Fig. 5). Further, temperature has previously been shown to correlate with seasonal mobility patterns, having important implications on disease transmission such as dengue and influenza^5,61^. Regardless, school holidays were found to be the most important predictor of mobility in our analyses, and represent an important factor for non-pharmaceutical interventions (such as extended school holidays and lockdowns) in curbing the spread of COVID-19 or other transmittable disease including influenza. While the number and timing of school holidays will vary by country, other studies have found that seasonal patterns of both domestic and international travel were consistent with public and school holidays across more than 90 countries^46^.

Recent studies using the mobility data used in these analyses have demonstrated that mobility can robustly predict disease spread (or a reduction in disease spread), and are on par with more comprehensive travel surveys such as commuter surveys or GPS devices^7,14,20^. Regardless, these data can require extensive technical expertise to work with, and can be difficult to obtain due to privacy concerns and anonymity. Further, many countries and populations may not be represented by mobile phone data due to sociodemographic biases. Towards this, openly available and freely accessible data estimating human mobility are increasingly being released, predominantly in response to the need for such data in understanding the effectiveness of control strategies in containing pandemic spread^24^. Here, we aim to demonstrate that a suite of standardly available covariates can be used to proxy changes in mobility patterns within LMIC settings using broadly and freely available data. While these covariates do not explain all the variance in observed mobility patterns, they represent an important set of predictors which can be used in combination with less comprehensive but more readily available mobility data, such as air travel statistics^62^. Similar models can be easily scaled to other LMICs where mobility data from mobile phones are lacking, and are particularly timely to establish baseline patterns of mobility as the global coronavirus pandemic subsides and travel returns to a new normal. For example, researchers have used novel and openly available data sources to study changes in mobility in Europe as a result of the pandemic^24^. Often these datasets represent only mobility over the course of the pandemic (e.g., 2020), however, representing the need for openly available data before and after COVID-19. Quantifying mobility patterns pre- and post-pandemic is vital in understanding when and whether mobility has returned to typical patterns seen pre-pandemic, or if mobility has indeed been permanently altered moving into the future.

### Limitations

These results should be interpreted in light of several important limitations. First, the Google mobility data is limited to smartphone users who have opted in to Google’s Location History feature, which is off by default. These data may not be representative of the population as whole, as biases in mobile phone ownership exist, and furthermore their representativeness may vary by location. Further, while the Google Aggregated Mobility Research Dataset represents movement at high spatial resolutions, our analyses are performed at the county level, and do not utilize this spatial granularity. Future work should explore the role of spatial resolution on mobility predictors, and whether some covariates are useful at some spatial scales, but not others. Importantly, these limited data are only viewed through the lens of differential privacy algorithms, specifically designed to protect user anonymity and obscure fine detail. Moreover, comparisons across rather than within locations are only descriptive since these regions can differ in substantial ways.

Further, these analyses are also subject to limitations inherent to the geospatial data and statistical modelling framework used. Firstly, because movement data are aggregated to county levels, it is not possible to identify the sociodemographic characteristics of the actual individuals moving. While this aggregation is crucial to protect confidentiality in analyses, it represents a limitation for these analyses, as individual or household survey data are not linked to mobility data, and therefore may not be representative of the population moving. Future research should aim to link individual movement patterns with sociodemographic questionnaires, where possible, or explore mathematical and statistical models linking these datasets. Towards this, the socioeconomic and geospatial covariates used in these analyses are often temporally lagged, and may not adhere exactly with the mobility data used. However, we aimed to mitigate this limitation by utilizing data aggregated at the monthly level. Lastly, these analyses are limited to the study region, and while they potentially represent major urban sub-Saharan hubs well (such as Nairobi), they may not be representative of other study countries or regions, particularly outside of Africa. Future work will aim at upscaling these modelling efforts to include additional sub-Saharan countries across the continent.

## Conclusions

How, when and where people move has important implications across disciplines, from urban planning to health and disease transmission^7,46^. The data used in these analyses represent movement before the global coronavirus pandemic emerged in the early months of 2020, and therefore are important for establishing baseline movement patterns in a non-pandemic setting. Future research should explore how seasonal movement patterns reduced or altered as a result of the pandemic, specifically within these study settings. Further, while previous research has demonstrated that human populations move predictably along seasonal gradient^2,4^, little has been done to quantify variability in these movement patterns at fine temporal and spatial scales, primarily owing to a lack of data spanning long periods of time at high spatial resolutions^14,30^.

Here, we used passively collected mobile phone data to describe seasonal movement patterns in Kenya across 2018 and 2019. We found that movement tended to peak in August and late December, strongly corresponding with school holidays. We found that neighbouring countries and provinces were highly interconnected, with primary international destinations in the Eastern African region surrounding Kenya, and primary domestic destinations between the Nairobi, Central and Eastern provinces. We further found that the number of school holidays was the strongest correlate of human mobility, followed by sociodemographic factors such as education, poverty, and accessibility to the nearest city. These results have important implications as the world returns to post-coronavirus movement patterns, particularly in monitoring and surveillance efforts of disease transmission.

## Supplementary Information

Supplementary Figures.
