# Supplementary material for: Practical geospatial and sociodemographic predictors of human mobility
Source: Sci Rep. 2021 Jul 28;11:15389. doi: 10.1038/s41598-021-94683-7 (PMC8319369; doi:10.1038/s41598-021-94683-7)
Supplement: Supplementary file 1 — Supplementary Figures. [file 41598_2021_94683_MOESM1_ESM.docx]

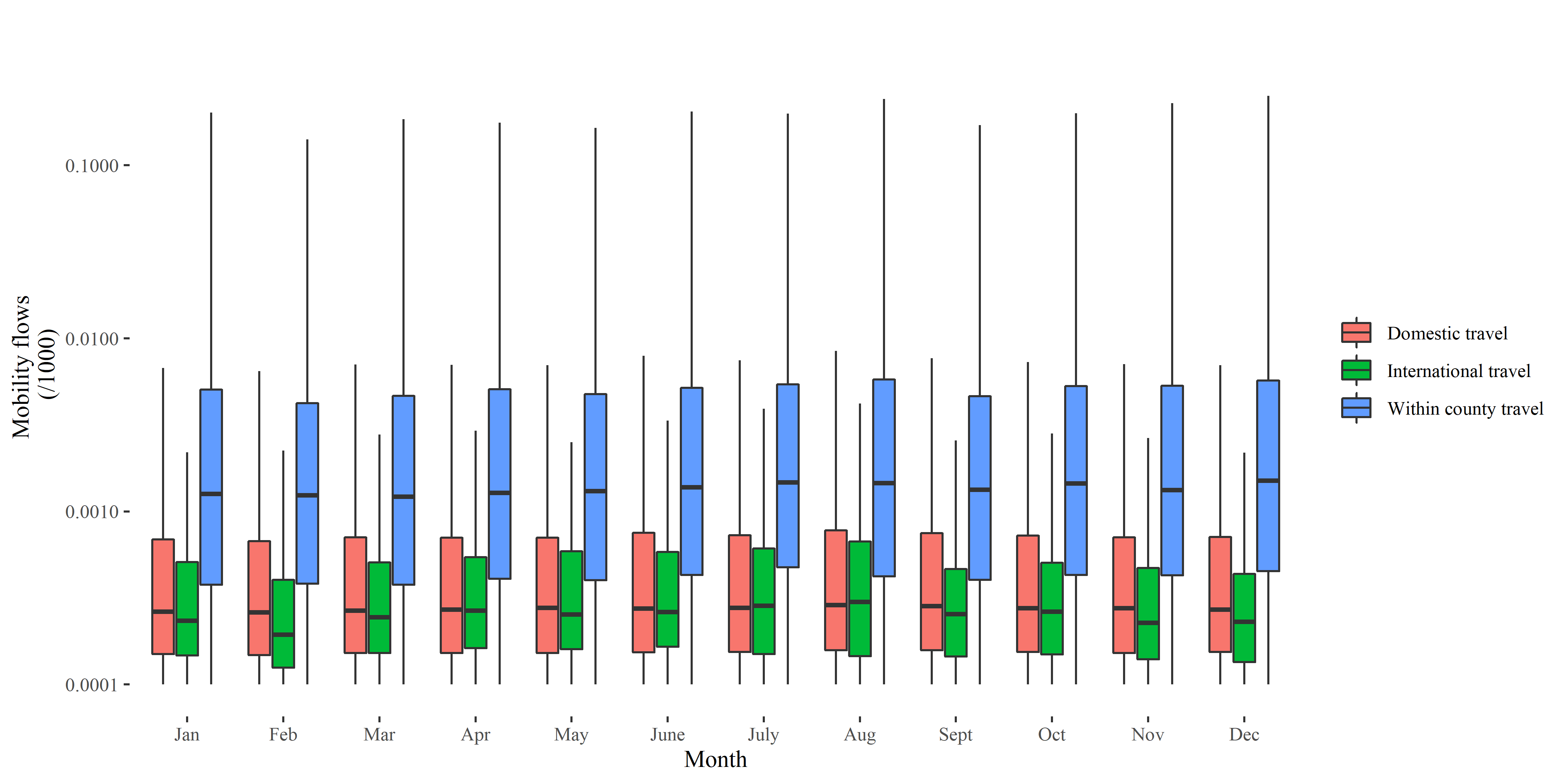
Figure S1. Box plots of monthly relative population flows by type of travel, as determined by origin/destination pairs. Red represents domestic travel within Kenya, green represents international travel from Kenya, and blue represents within-county travel. Months represent average population flows over 2018 and 2019.


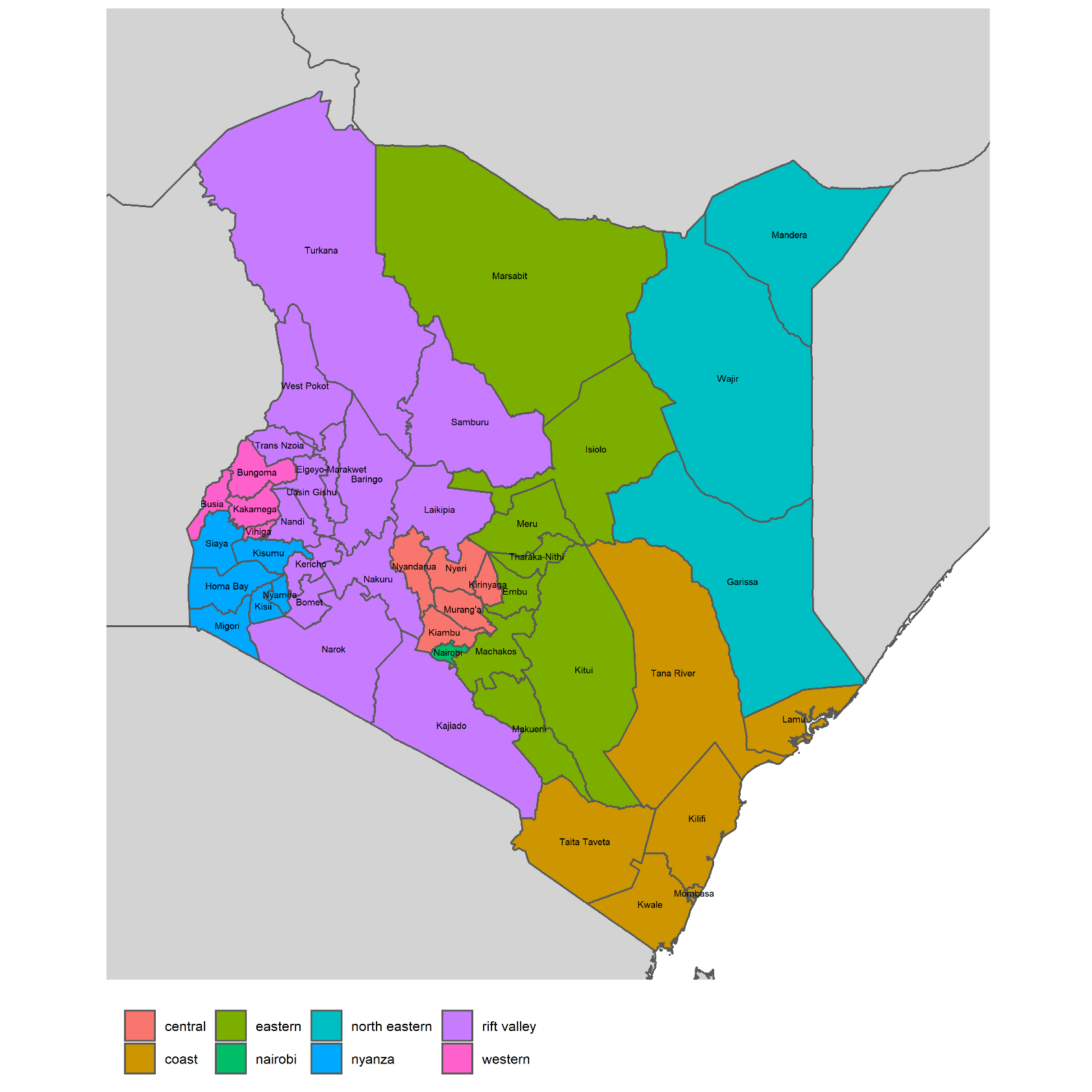


Figure S2. Counties in Kenya by province. R software, version 4.0.4, https://www.r-project.org/.


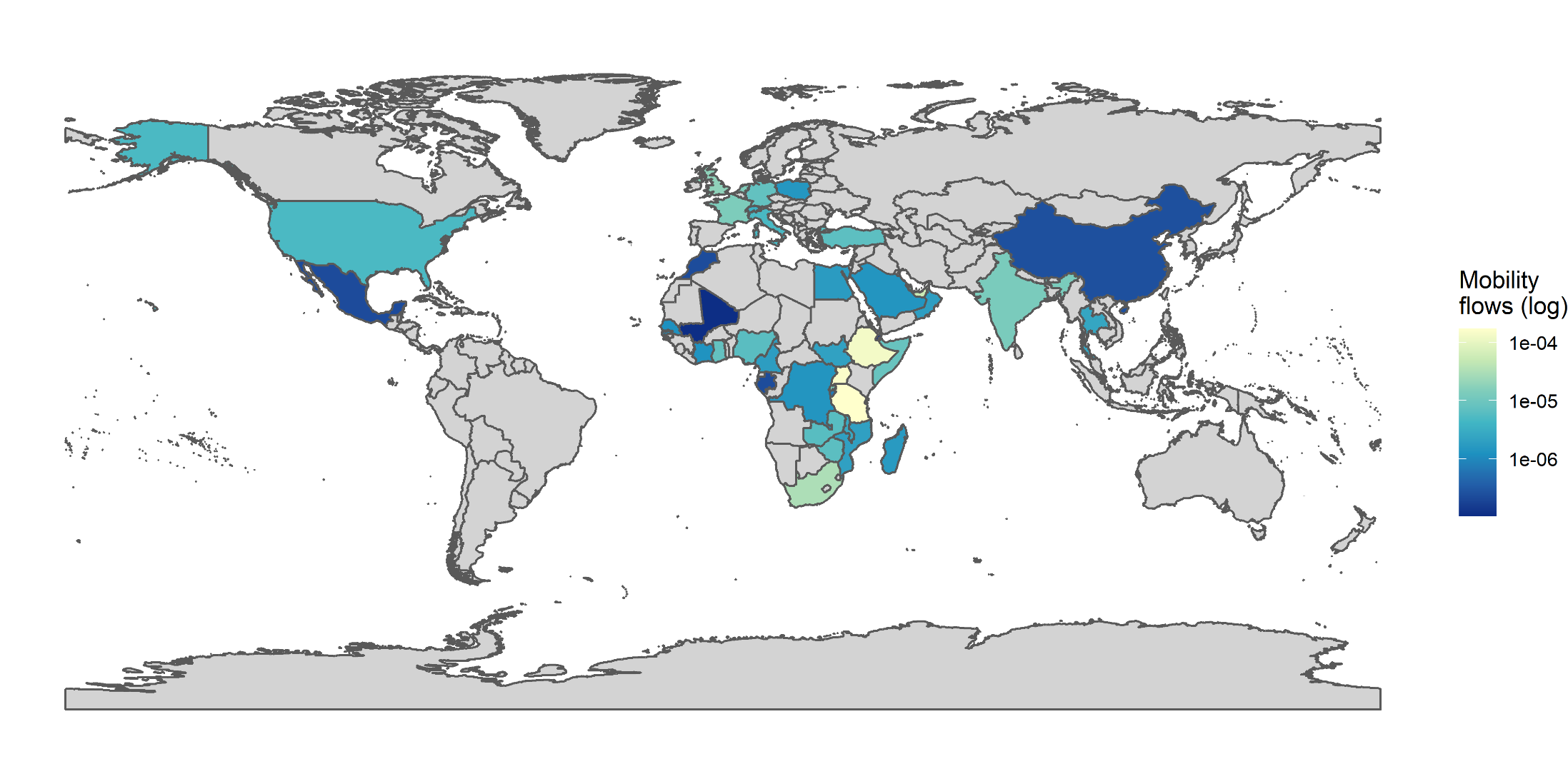
Figure S3. Total international mobility flows from Kenya over 2018 and 2019. Flows were log transformed to highlight spatial heterogeneity. R software, version 4.0.4, https://www.r-project.org/.


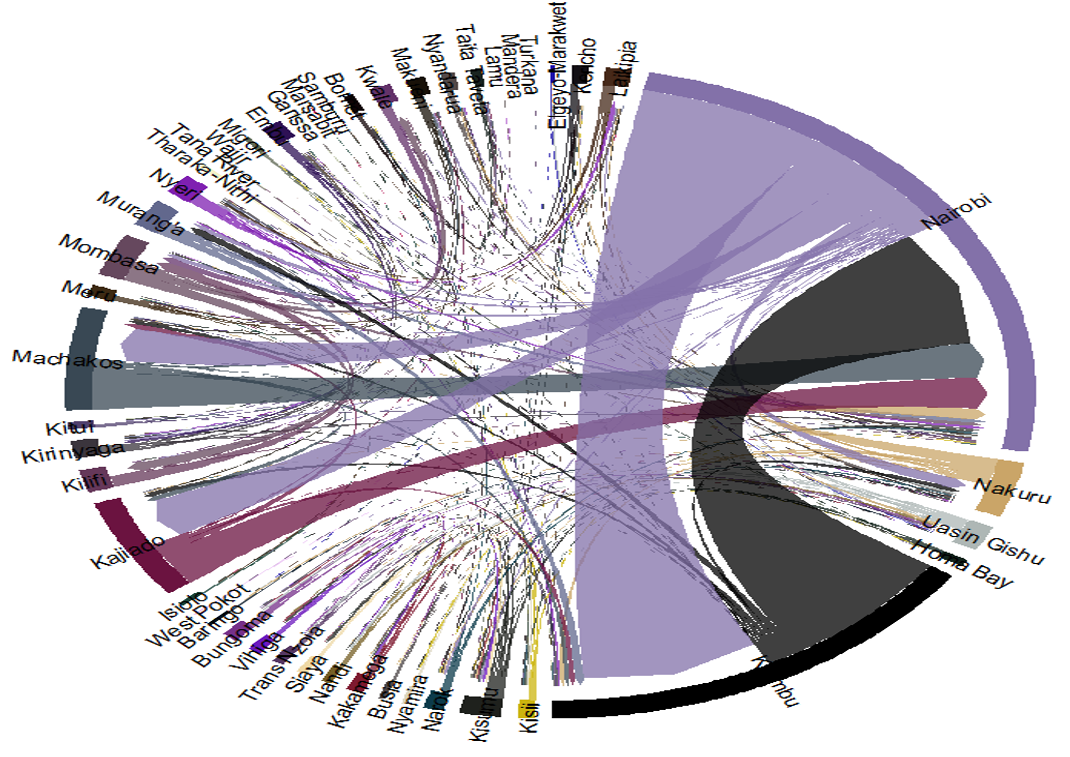


Figure S4. Bidirectional domestic mobility patterns between Kenya counties. Arrows indicate directionality of movement.
